# Evaluation of partial resheathing of EmboTrap III using the microcatheter (PREMIER) technique for fibrin-rich hard clots in an *in vitro* vessel model

**DOI:** 10.3389/fneur.2024.1368890

**Published:** 2024-08-07

**Authors:** Satoshi Namitome, Yoichiro Nagao, Yuya Shigehatake, Junichi Matsuo, Keisuke Kawamoto, Kenji Kuroki, Hirotaka Hayashi, Makoto Nakajima, Tadashi Terasaki, Mitsuharu Ueda, Seigo Shindo

**Affiliations:** ^1^Department of Neurology, Japanese Red Cross Kumamoto Hospital, Kumamoto, Japan; ^2^Department of Neurology, Kumamoto University, Kumamoto, Japan; ^3^Department of Neurology, Saiseikai Kumamoto Hospital, Kumamoto, Japan; ^4^Department of Neurology, Kagoshima City Hospital, Kagoshima, Japan

**Keywords:** mechanical thrombectomy, large vessel occlusion, stroke, hard clots, *in vitro* vessel model

## Abstract

**Background and purpose:**

Despite the ongoing advancements in mechanical thrombectomy for large vessel occlusions causing acute ischemic stroke, successful recanalization is not achieved in all patients. One contributing factor is the presence of fibrin-rich hard clots. We proposed a new technique called the PREMIER technique, which aims to retrieve fibrin-rich clots. This study evaluated the efficacy of the PREMIER technique on fibrin-rich and erythrocyte-rich clots by comparing it with the simple use of EmboTrap III in an *in vitro* vessel model.

**Methods:**

The PREMIER technique involves partially resheathing a fully deployed EmboTrap III (CERENOVUS, Johnson & Johnson Medical Devices, Irvine, California, USA) using a microcatheter to capture and retrieve a hard clot between the inner channel and outer cages of EmboTrap III. We compared recanalization rate of the PREMIER technique with the simple use of EmboTrap III in an *in vitro* vessel model, occluding the M1 segment with fibrin-rich hard clots (0% erythrocyte composition) and erythrocyte-rich clots (50% erythrocyte composition).

**Results:**

Among the 40 procedures (10 each for the PREMIER technique and the simple use of EmboTrap III for two different clots) for fibrin-rich clots, the PREMIER technique achieved successful recanalization in all 10 cases, with a significantly higher recanalization rate than the EmboTrap III (100% vs. 50%, *p* = 0.03). For erythrocyte-rich clots, the recanalization rate was not significantly different in the PREMIER technique compared with the simple use of EmboTrap III (80% vs. 70%, *p* = 1.00).

**Conclusion:**

The PREMIER technique is a novel technique for acute large-vessel occlusions caused by fibrin-rich hard clots that hinders successful recanalization during mechanical thrombectomy.

## Introduction

Mechanical thrombectomy (MT) with a stent retriever or aspiration catheter for acute large-vessel occlusion has become the standard therapy. Many methods have been proposed for MT, including the combined use of a stent retriever and an aspiration catheter (1–4). Moreover, several randomized clinical trials have been conducted to compare the efficacy of stent retrievers alone with aspiration catheters or combination techniques (5–7). However, the most effective method remains undetermined. Additionally, the methods used have shown a high successful recanalization rate; however, approximately 8–17% of the patients do not achieve successful recanalization.

Despite advances in thrombectomy methods and devices, the occlusion of vessels caused by fibrin-rich hard clots remains a potential factor contributing to the lack of successful recanalization. Fibrin-rich clots are associated with lower recanalization rates and require a higher number of passes compared to erythrocyte-rich clots (8–10).

Therefore, we proposed a new method to retrieve fibrin-rich hard clots called the Partial Resheathing of EmboTrap III (CERENOVUS, Johnson & Johnson Medical Devices, Irvine, California, USA) using the MIcrocathetER (PREMIER) technique.

In this study, we evaluated the efficacy of the PREMIER technique on hard and soft clots by comparing the PREMIER technique with the simple use of EmboTrap III in an *in vitro* vessel model in which the M1 segment of the middle cerebral artery was occluded by fibrin-rich hard and erythrocyte-rich soft clots.

## Materials and methods

### Device description

**Figure 1:**
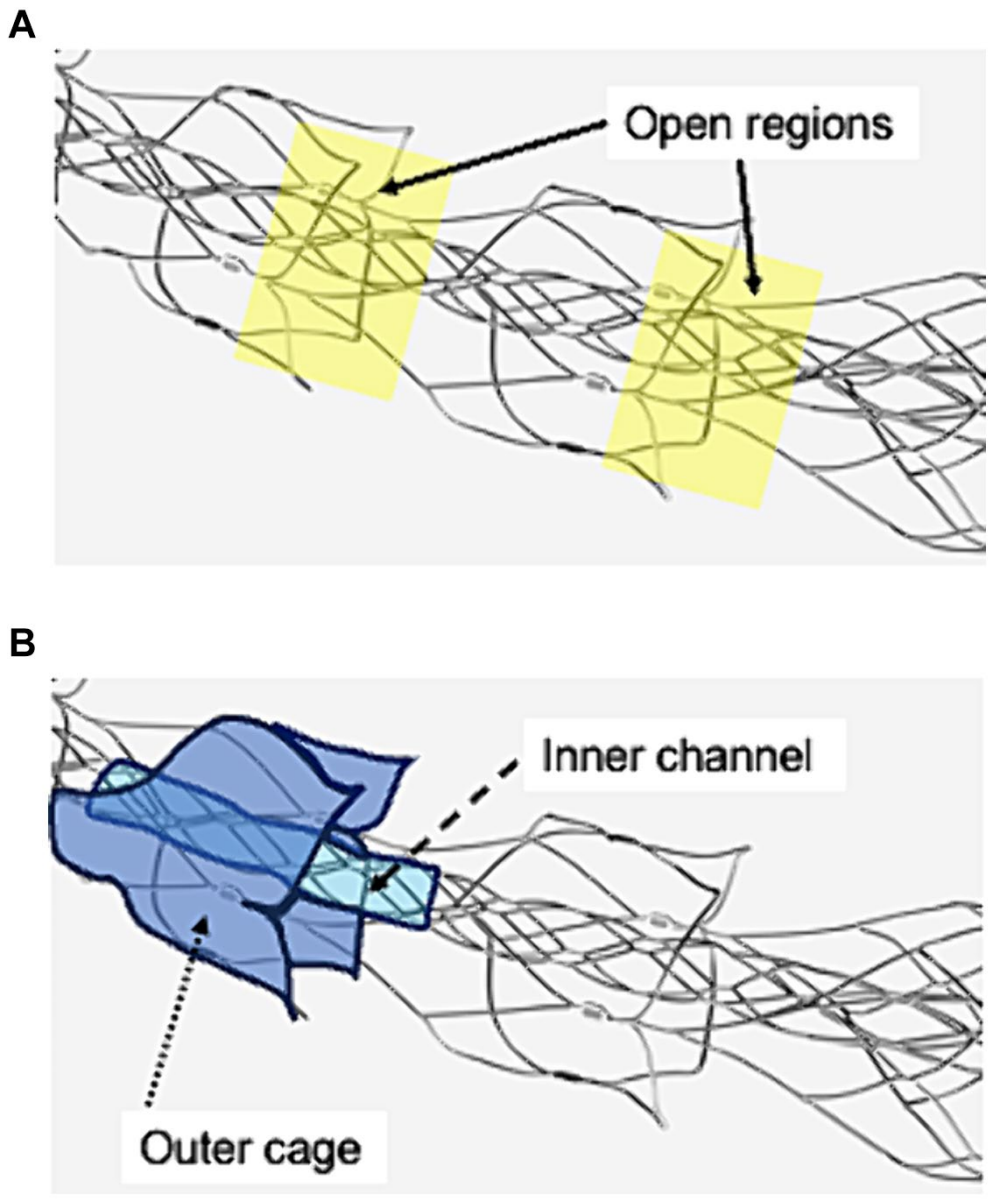
Visualization of the open regions, outer cage, and inner channel of EmboTrap III with markers. **(A)** Open regions are between EmboTrap III’s outer cage and the distal outer cage. **(B)** EmboTrap III’s inner channel and outer cage.

All procedures were performed using an 8Fr guiding catheter (Asahi-Intec, Aichi, Japan), a 0.021-inch microcatheter (Medtronic, Tolochenaz, Switzerland), a 0.014-inch microguidewire (Asahi-Intec), and EmboTrap III (Cerenovus, Johnson & Johnson, New Brunswick, NJ, USA). There are open regions between its outer and the distal outer cages (Figure 1A). EmboTrap III has an inner channel and outer cages (Figure 1B).

### Vascular model and clot description

The vascular silicone model was made following an original design to faithfully reproduce a unilateral cerebral vascular flow (Elastrat Sàrl, Geneva, Switzerland). This model included the right internal carotid artery (ICA), right middle cerebral artery (MCA; including the M1 and M2 segments), right anterior cerebral artery (ACA; including the A1 segment, bilateral A2 segment and anterior communicating artery), right vertebral artery, basilar artery, and right posterior communicating artery. Additionally, the petrous portion of the ICA is 5.5–6.0 mm, the cavernous portion of the ICA is 6.0 mm, the top of ICA is 4.0 mm, and the M1 and A1 segments have a diameter of 3.0 mm. The model was connected to a pulsatile pump system (FlowTek 125; United Biologics Inc., Santa Ana, CA, USA). The circulating water temperature and pressure were maintained at 37°C and approximately 150 mmHg, respectively, and they were frequently monitored (M-382, As-one, Osaka, Japan).

Fibrin-rich hard and erythrocyte-rich soft clots were produced using porcine blood according to the previously reported method by Duffy et al. (11) Furthermore, fibrin-rich hard clots, with 0% erythrocyte composition, and erythrocyte-rich soft clots, with 50% erythrocyte composition, were obtained. These clots were cut into 5 ± 0.5 mm × 10 ± 0.5 mm × 17 mm in size to create an occluded vessel model in the M1 segment.

### Description of the procedures

**Figure 2:**
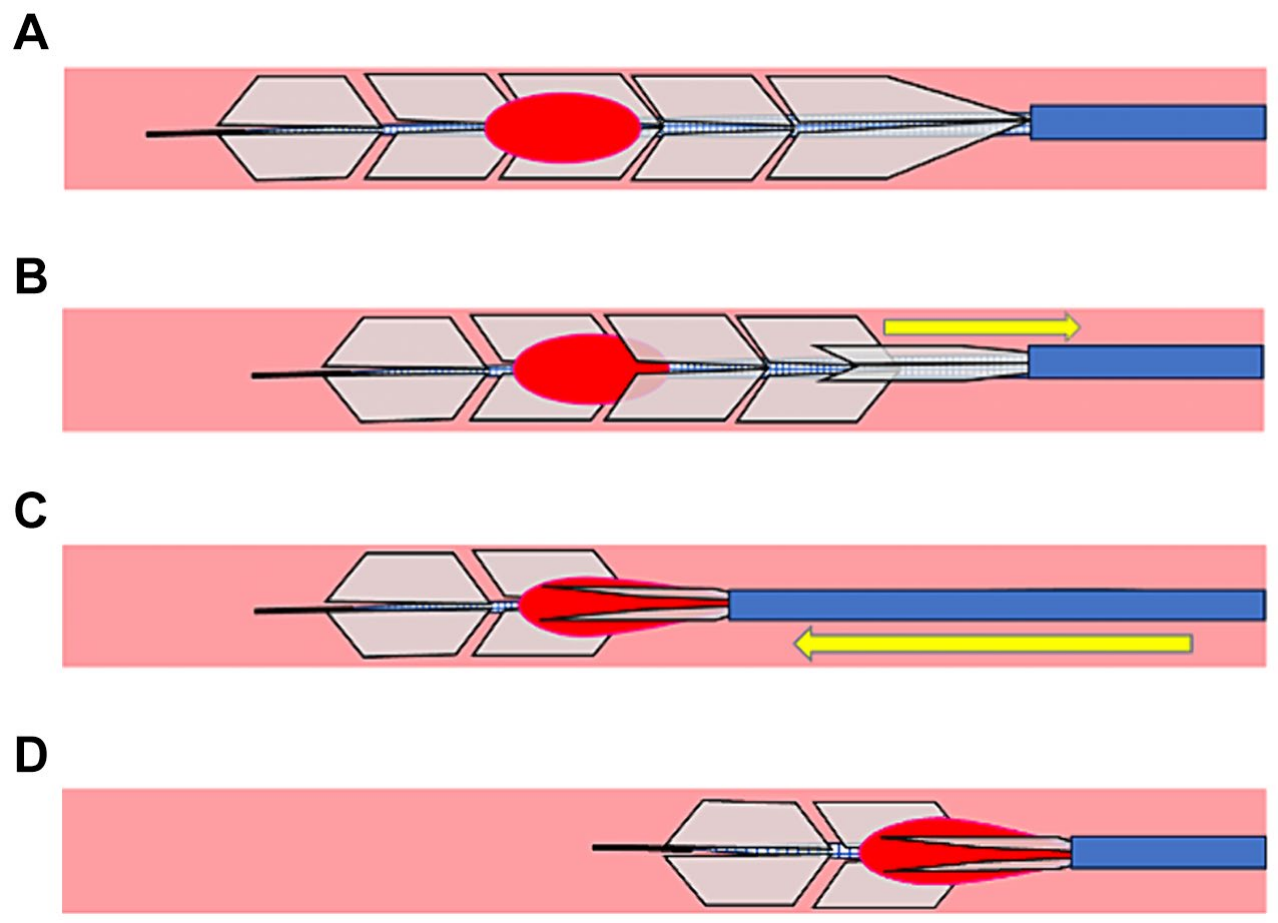
Partial resheathing of EmboTrap III with the microcatheter (PREMIER) technique. Diagram of the *in vitro* experiment and a corresponding illustration example. **(A)** EmboTrap III is fully deployed in the clot. **(B)** EmboTrap III is pulled slightly. **(C)** EmboTrap III is partially resheathed using the microcatheter until resistance is experienced. **(D)** EmboTrap III and the microcatheter are withdrawn together as units.

An 8Fr guiding catheter was placed in the cervical ICA. The microcatheter is navigated over a microguidewire distal to the clot. Moreover, EmboTrap III was deployed to cover the distal and proximal ends of the clot. When thrombectomy was performed with the simple use of EmboTrap III, EmboTrap III was maintained in this position for 2 min (Figure 2A) and retrieved into the guiding catheter at a rate of 2 cm/s. When the PREMIER technique was performed, EmboTrap III was pulled slightly after deployment to trap the clot core in the open region (Figure 2B). Subsequently, EmboTrap III was partially resheathed with the microcatheter until resistance was experienced (Figure 2C). Therefore, a clot was trapped between the inner channel and outer cages of EmboTrap III. While maintaining this position, EmboTrap III and the microcatheter were withdrawn together as units into the proximal guiding catheter at 2 cm/s for clot retrieval (Figure 2D).

To avoid arbitrary manipulation of either procedure, both procedures were performed without observing the vascular model after EmboTrap III was deployed. Moreover, two neurointerventionalists performed mechanical thrombectomy. Each neurointerventionalists performed five each of the PREMIER technique and the simple use of EmboTrap III, on the vessel model of the M1 segment occlusion with two clot types. If occlusion by a fibrin-rich hard clot failed to recanalize with the simple EmboTrap, a rescue treatment was performed to retrieve the clot using the PREMIER technique (rescue cases).

### Measurements and outcomes

Successful reperfusion was defined when the M1 occlusion was recanalized and there was no obvious occlusion in the distal vessel, as seen by naked eye; otherwise, it was defined as a failed recanalization. Distal emboli were collected, including procedures that failed to recanalize. A cell strainer attached to a Thrombuster III (Kaneka medix, Osaka, Japan) was placed at the entrance of the tank where water circulating through the vessel model was returned by a pulsatile pump system, which could collect emboli greater than 70 μL. The cell strainer was checked for distal embolization at the end of each procedure. The primary outcome of this study was successful recanalization rate. In one case, in which a fibrin-rich hard clot was retrieved by the PREMIER technique, non-contrast computed tomography (CT) (Canon, Otawara, Tochigi, Japan) was performed using a 320-section multidetector scanner to confirm the location of the outer cage, the inner channel of EmboTrap III, and the fibrin-rich hard clot. This was done while EmboTrap III was partially resheathed in a clot retrieval state.

### Analysis

We compared the frequency of successful recanalization between thrombectomy with the PREMIER technique and thrombectomy with EmboTrap III using Fisher’s exact test. Statistical significance was set at *p* < 0.05. Statistical analyses were performed using the JMP 10.1 (SAS Institute Inc., Cary, NC, USA).

## Results

### Successful revascularization and distal emboli *in vitro* vessel model

Forty procedures were performed (10 each) using the PREMIER technique and EmboTrap III in an *in vitro* vessel model, occluding the M1 segment with fibrin-rich hard clots (0% erythrocyte composition) and erythrocyte-rich clots (50% erythrocyte composition). In cases where the vessel model was occluded by fibrin-rich hard clots, all 10 cases using the PREMIER technique were successfully recanalized, whereas only 5 of 10 cases achieved successful recanalization with the simple use of EmboTrap III (100% vs. 50%, *p* = 0.03) (Table 1). In the five cases where the simple use of EmboTrap III failed and the PREMIER technique was used as a rescue treatment, successful recanalization was achieved in all cases (*p* = 0.10). Conversely, in cases where the vascular model was occluded by erythrocyte-rich soft clots, recanalization was achieved in eight cases with the PREMIER technique and in seven cases with the simple use of EmboTrap III, with no statistically significant difference in the recanalization rates (*p* = 1.00).

**Table 1:** Comparison of outcomes by the PREMIER technique and the simple use of EmboTrap III.

Variable	Simple use of EmboTrap III (*n* = 10)	PREMIER technique (*n* = 10)	*p*-value	Rescue case (*n* = 5)	*p*-value
Fibrin-rich hard clot					
Success recanalization, *n* (%)	5 (50%)	10 (100%)	0.03	5 (100%)	0.10
Distal emboli, *n* (%)	0 (0%)	0 (0%)	-	0 (0%)	-
Erythrocyte-rich soft clot					
Success recanalization, *n* (%)	7 (70%)	8 (80%)	1.00	-	-
Distal emboli, *n* (%)	2 (20%)	1 (10%)	1.00	-	-

Distal emboli did not occur in fibrin-rich hard clots using either the PREMIER technique or the simple use of EmboTrap III. In erythrocyte-rich soft clots, distal emboli occurred in only one case with the PREMIER technique and in two cases with the simple use of EmboTrap III, but the difference was not statistically significant.

### Observation of the devices after retrieval of the hard clot using the PREMIER technique

**Figure 3:**
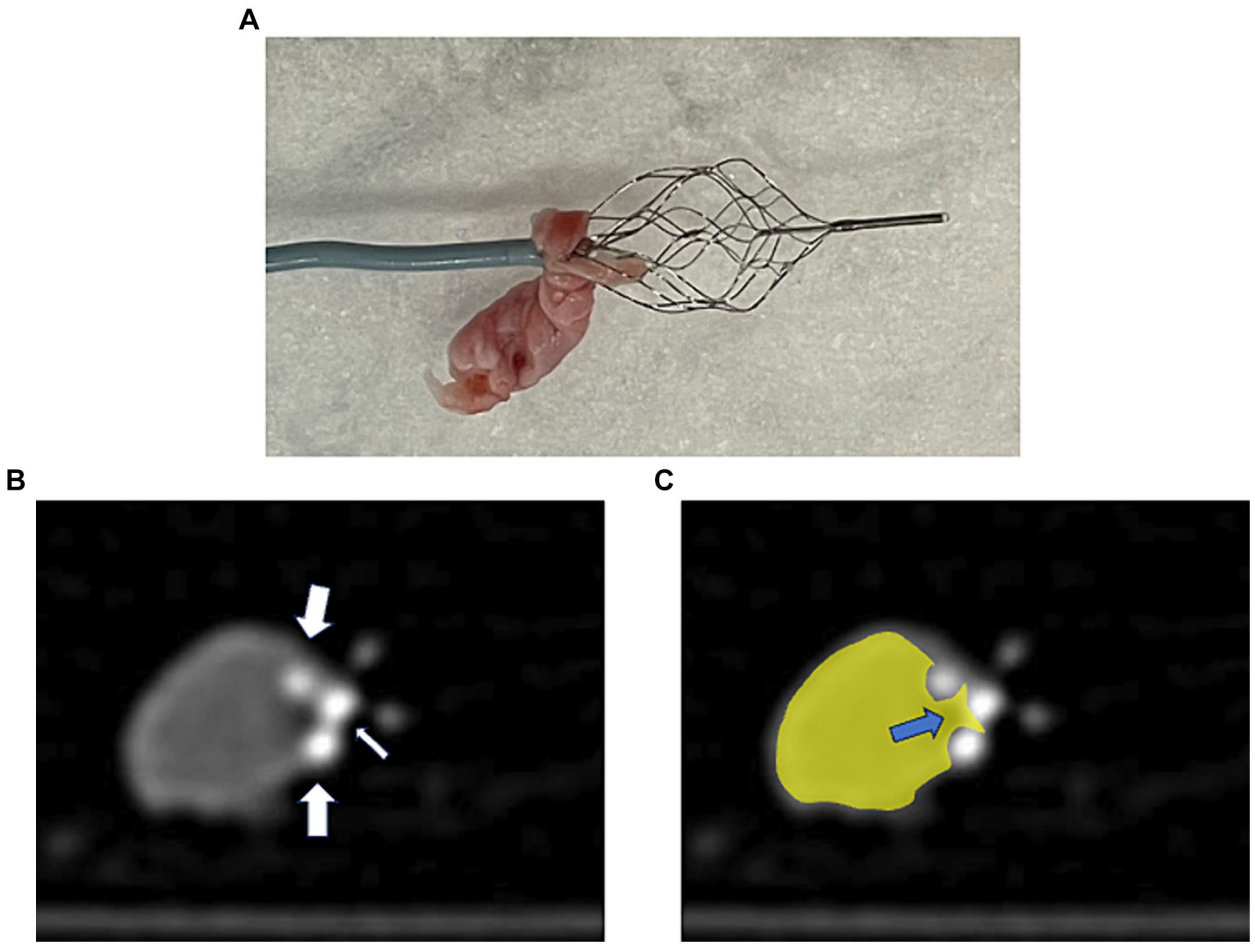
A case of thrombectomy with the PREMIER technique. **(A)** Actual fibrin-rich hard clot retrieved with the PREMIER technique. **(B)** Plain computed tomography image of fibrin-rich hard clot and EmboTrap III following the PREMIER technique. The big arrows point to the outer cage and small arrow points to the inner channel of EmboTrap III. **(C)** Colored version of the figure **(B)**. The hard clot is colored yellow. The blue arrows indicate the section where the clot is pinched between the inner channel and the outer cage of EmboTrap III.

Upon observing the devices after a fibrin-rich hard clot retrieval using the PREMIER technique, a part of the fibrin-rich hard clot was located between the microcatheter and EmboTrap III (Figure 3A). Furthermore, computed tomography after clot retrieval in a randomly selected case, it showed that the inner channel and outer cages of EmboTrap III (Figure 3B) and a part of the hard clot was pinched between the inner channel and outer cage, which was closed by the microcatheter (Figure 3C).

## Discussion

We propose a new technique called the PREMIER technique, which is effective for fibrin-rich hard clots in thrombectomy. This technique involves resheathing EmboTrap III with a microcatheter, pinching the hard clots between the inner channel and outer cages of EmboTrap III. Computed tomography also revealed that the hard clot was trapped between the inner channel and outer cage of the resheathed EmboTrap III.

Thrombectomy for high density clots on no-contrast CT, indicating erythrocyte richness, has reported the efficacy of stent retrievers, while low density clots (<50 Hounsfield Units) is reported to achieve relatively higher reperfusion rates with contact aspiration (12, 13). Mohammaden et al. (13) reported that erythrocyte-rich clots are more easily deformable than fibrin-rich clots, allowing the stent retriever struts to penetrate more easily in erythrocyte-rich clots. Conversely, fibrin-rich clots were less deformable. In such cases, contact aspiration minimalized the attenuation of device-clot interaction forces by concentrating the retrieval force along the clot’s shorter axis and closely aligning it with the direction of movement. As a result, contact aspiration effectively captures the clot like a cork and harder clots can be retrieved more effectively.

On the other hand, there are negative aspects associated with contact aspiration. it has been reported that an aspiration catheter causes distal embolism more frequently in a procedure than a stent retriever or during the combined use of a stent retriever and an aspiration catheter (14, 15). Furthermore, Bala et al. reported that contact aspiration was associated with a decreased likehood of first pass effect with higher vessel tortuosity, and an increasing number of passes with longer clots (16). They suggested that this result was associated with the bending or collapse of the lumen of the aspiration catheter in tortuous arterial segments, significant reduction in suction force, and misalignment between the aspiration catheter and the clot axis.

The PREMIER technique is optimal for retrieving fibrin-rich hard clots, which are difficult to remove using conventional stent retriever techniques. Additionally, this technique is thought to have a smaller impact from vessel tortuosity compared to contact aspiration. In such cases, as long as the microcatheter can guide to the distal part of the clot, it is capable of strongly trapping the fibrin-rich hard clot. Hard clots can be trapped distally to the open region of EmboTrap III. In cases with vessel tortuosity, intentionally shortening the deployed length of EmboTrap III may reduce vessel shift and bleeding complications.

To overcome the aforementioned weaknesses of stent retrievers and aspiration catheters, new devices and methods for retrieving hard clots have been developed. For example, a new stent retriever called Nimbus (Cerenovus) has been designed to retrieve fibrin-rich hard clots (17). Moreover, Lehnen NC et al. (18) reported a good recanalization rate with Nimbus in patients in whom recanalization with conventional techniques had failed. However, that study suggested that Nimbus could be used as a second-line device because it was not designed to retrieve erythrocyte-rich soft clots and was more expensive than other stent retrievers. Therefore, Nimbus was used as a second-line device after two failed attempts using standard stent retrievers.

Some reports suggested a hyperdense artery sign on non-contrast CT or a susceptibility vessel sign on magnetic resonance imaging can be used to identify erythrocyte-rich clots (19, 20). However, it is not easy to distinguish reliably fibrin-rich clots from erythrocyte-rich clots in actual clinical setting.

In this *in vitro* study, the PREMIER technique showed distal embolization in erythrocyte-rich clots. When resheathing EmboTrap III with the microcatheter, there is a risk of oversheathing it without experiencing resistance, potentially causing fragmentation of fragile erythrocyte-rich clots. Therefore, the PREMIER technique may be recommended when mechanical thrombectomy by the conventional technique fail to achieve successful recanalization and presence of hard clots is suspected. However, the PREMIER technique allows additional procedure using only EmboTrap III without additional new devices, that is considered superior to Nimbus and the combined technique in this regard.

This study has some limitations. First, it was performed using an original unilateral *in vitro* vascular model without the Willis arterial circle. This did not allow complete reproduction of normal cerebral circulation. Second, this study was not performed under digital subtraction angiography, but was performed under visual observation until EmboTrap III was deployed in a clot. Feasibility, required time, or other potential challenges of the procedures in the clinical angiography system should be verified. Third, aspiration catheters and the combined use of stent retrievers and aspiration catheters were not compared. Further studies that compare these techniques are required. Finally, this study on the PREMIER technique was conducted using a small number of *in vivo* cases. Further research is required to evaluate its efficacy and safety in a clinical setting.

## Conclusion

Our *in vitro* vessel model assessment suggested that the PREMIER technique is effective for acute large-vessel occlusions caused by fibrin-rich hard clots which can hinder successful recanalization during mechanical thrombectomy.

## Data availability statement

The original contributions presented in the study are included in the article/supplementary material, further inquiries can be directed to the corresponding author.

## Author contributions

SN: Conceptualization, Data curation, Formal analysis, Investigation, Writing – original draft, Methodology, Visualization. YN: Conceptualization, Writing – review & editing. YS: Conceptualization, Writing – review & editing. JM: Writing – review & editing. KKa: Writing – review & editing. KKu: Writing – review & editing. HH: Writing – review & editing. MN: Writing – review & editing. TT: Writing – review & editing. MU: Writing – review & editing. SS: Conceptualization, Data curation, Investigation, Methodology, Supervision, Writing – original draft, Writing – review & editing.
